# The respiratory microbiome influences the occurrence of respiratory diseases in children

**DOI:** 10.3389/fped.2026.1734912

**Published:** 2026-05-29

**Authors:** Kui Zheng, Shu hong Zhou, Shunli Liao, Jing Mu, Zhou Bo Han, Bo Wang

**Affiliations:** 1 Department of Paediatrics, Bishan Hospital of Chongqing Medical University (Bishan Hospital of Chongqing), Chongqing, China; 2 Chongqing Business Vocational College, Chongqing, China

**Keywords:** children, gut-lung axis, respiratory flora, respiratory infection, respiratory microbiome

## Abstract

Respiratory diseases are common and frequently occurring illnesses in children. In recent years, studies have confirmed that the respiratory microbiome is closely associated with the susceptibility and severity of respiratory diseases in children, and its microbial composition and metabolites are involved in the occurrence and development of respiratory infections, allergic airway inflammation, asthma and other disorders. Based on the cutting-edge research findings of the past 2–3 years, this review systematically summarizes the composition and sources of the respiratory microbiome in children, the characteristics of its colonization and dysbiosis, the interaction mechanisms with the host immune system, as well as the correlation rules with common respiratory diseases. It also focuses on elaborating novel therapeutic strategies such as probiotic therapy, phage therapy, and antimicrobial peptide (AMP) therapy, aiming to provide new ideas and directions for the research on the pathogenesis and clinical precision treatment of childhood respiratory diseases.

## Introduction

Respiratory diseases remain one of the leading causes of high morbidity and mortality in children worldwide (1). Previous studies have indicated that genetic factors, immune function, environmental factors and other elements influence children's susceptibility to respiratory diseases (2).With the development and application of detection technologies such as next-generation sequencing (NGS), it has been gradually recognized that the human respiratory tract from the nasal cavity to the alveoli harbors a complex microbial community composed of bacteria, viruses, fungi and archaea, whose genomes and metabolites collectively constitute the respiratory microbiome (3). A healthy respiratory flora exerts a “colonization resistance” effect to inhibit the colonization of pathogenic bacteria by enhancing mucosal barrier function and promoting immune maturation (4, 5), while floral dysbiosis is closely related to the occurrence, development and prognosis of respiratory diseases in children (4).

Remarkable achievements have been made in the research of the intestinal microbiome. For instance, specific microbiome characteristics have been identified for intestinal diseases such as Crohn's disease (6). Fecal microbiota transplantation (FMT) has been proven to restore the composition of the intestinal microbiome and exert a significant therapeutic effect on the treatment of recurrent Clostridioides difficile infection (7). In contrast, the research on the pulmonary microbiome is still in its infancy due to the difficulty in obtaining sputum samples from the lower respiratory tract of healthy children. Meanwhile, technical limitations such as sample contamination, low microbial biomass, and lack of standardized protocols further hinder pediatric respiratory microbiome studies.The study of the respiratory microbiome can not only reveal the microecological pathogenesis of respiratory diseases but also provide a theoretical basis for the development of microecology-targeted therapeutic strategies (8).This narrative review aims to synthesize recent advances in the pediatric respiratory microbiome, clarify its role in common respiratory diseases, and evaluate emerging microecology-targeted therapeutic strategies.

## Composition and sources of the respiratory Microbiota

The respiratory tract is an important organ system responsible for O₂ and CO₂ exchange in the human body, which is divided into the upper respiratory tract (URT) and the lower respiratory tract (LRT) with the inferior border of the cricoid cartilage as the boundary (9). Microorganisms such as bacteria, archaea, viruses and fungi are widely distributed in the upper and lower respiratory tracts, and different anatomical sites have specific microbial communities and dominant flora, whose genomes and metabolites together form the microbiome (5, 10). The respiratory flora of healthy children is mainly composed of Firmicutes, Proteobacteria, Bacteroidetes, Actinobacteria and Fusobacteriota (11). The nasal cavity has the highest abundance of Firmicutes and Actinobacteria and the lowest of Bacteroidetes and Fusobacteriota, the tonsils and oropharynx are rich in Haemophilus and Neisseria (11). The richness and diversity of the upper respiratory flora in children increase with age, reach a peak in childhood, and gradually decline in adulthood (12, 13). Zelasko et al. (12) found significant differences in the composition of nasopharyngeal and oral flora between infants and young children (0–24 months) and adults (18–40 years): the nasal cavity of infants and young children is dominated by Moraxella and Dolosigranulum, with a relatively high content of opportunistic pathogens such as Haemophilus influenzae and Streptococcus pneumoniae, the nasal cavity of adults is mainly composed of Cutibacterium acnes and Corynebacterium, with a higher proportion of beneficial bacteria. Hernandez-Leyva et al. (13) further confirmed the age-related composition of the nasopharyngeal flora: 5 months to 6 years old, dominated by Dolosigranulum + Corynebacterium, 6 to 12 years old, the proportion of Staphylococcus aureus increases and becomes the dominant group, over 12 years old, the microbial diversity reaches a peak, dominated by mixed flora (including Corynebacterium, Streptococcus, Veillonella, etc.), as shown in Table 1. Most studies hold that the lower respiratory flora in children is mainly derived from the microaspiration and migration of the upper respiratory tract (oral cavity, nasopharynx) (8, 14), and its dynamic balance depends on the interaction between microbial migration, host clearance (cough, respiratory ciliary movement, macrophage phagocytosis, etc.) and microbial proliferation (13). The lower respiratory tract of healthy children is dominated by Streptococcus, Veillonella and Prevotella, and the microbial diversity is positively correlated with the state of pulmonary health (14). Viruses such as rhinovirus and bacteriophages, as well as fungi such as Candida, can also be detected in the respiratory tract of healthy children (2). The overall detection rate of respiratory viruses in healthy asymptomatic children is 67%, including rhinovirus, bocavirus, adenovirus and coronavirus (15), fungi usually form a symbiotic relationship with the host, and the pulmonary mycobiome acts as a cofactor of host immunity and inflammatory response, while having potential pathogenicity (2). At present, the specific functions of fungi and viruses in the homeostasis of the respiratory microecology remain unclear and need further in-depth study.

**Table 1:** Characteristics of dominant nasopharyngeal flora in children of different age groups.

Age group	Core microbial characteristics	Microbial diversity	Key interaction patterns
5 months to 6 years	Dominant flora: Dolosigranulum + Corynebacterium	Low to moderate α-diversity, slightly increasing with age	Dolosigranulum is positively correlated with Corynebacterium, both are negatively correlated with Staphylococcus aureus
	Minor flora: a small amount of Streptococcus and Staphylococcus		
6 to 12 years	Dominant flora: Staphylococcus aureus	α-diversity is slightly higher than that in the previous stage	Staphylococcus aureus is dominant and inhibits the proliferation of other beneficial bacteria
	Minor flora: Dolosigranulum and Corynebacterium (decreased proportion)		
12 to 18 years	Dominant flora: mixed flora (Corynebacterium, Streptococcus, Veillonella, Prevotella, etc.)	α-diversity reaches the peak in childhood	Multiple bacterial taxa coexist synergistically with Streptococcus, which serves as the core genus. Streptococcus is positively correlated with oral-derived microbiota and exhibits negative correlations with opportunistic pathogens such as Staphylococcus aureus and Moraxella

## Colonization and dysbiosis of the respiratory Microbiota

### Colonization and colonization resistance

The colonization of the respiratory microbiota starts at the time of neonatal birth, and the initial colonization pattern is significantly affected by factors such as delivery mode, breastfeeding, antibiotic therapy, and viral infection (16). Infants and young children delivered vaginally and/or breastfed have airway dominant flora mainly composed of Lactobacillus, Corynebacterium and Dolosigranulum, which helps reduce the risk of respiratory tract infections in infants and young children (16, 17), asymptomatic viral infection in the neonatal period can promote the colonization of pathogenic bacteria such as Moraxella and Haemophilus, increasing the risk of recurrent respiratory tract infections or wheezing in children at a later stage (4). In the dynamic balance of the respiratory microecology, the symbiotic relationship between the host and microorganisms can effectively inhibit the excessive proliferation of pathogenic bacteria, a phenomenon known as “colonization resistance” (2, 18). Potential pathogenic bacteria such as Haemophilus influenzae, Streptococcus pneumoniae and Moraxella catarrhalis are common in asymptomatic colonizers, and their colonization rate is often higher than the infection rate (18). For example, the colonization rate of Haemophilus influenzae in healthy children is as high as 30%–88%, and only a small number of colonized children develop clinical infections (18).

A variety of microorganisms in the healthy respiratory tract can provide anti-infection protection for the host by directly inhibiting the excessive proliferation of pathogenic bacteria and secreting antibacterial substances such as bacteriocins to prevent the formation of pathogenic bacterial biofilms, and at the same time promote immune tolerance and maturation in the pulmonary microenvironment (4, 8). For instance, Rothia in the human nasal cavity can inhibit the colonization of Moraxella catarrhalis by secreting the peptidoglycan endopeptidase SagA (19), Corynebacterium competes directly with Staphylococcus aureus for nutrients and adhesion sites on the surface of the skin and mucous membranes, limiting its reproduction (4, 20), Streptococcus salivarius, Dolosigranulum and Corynebacterium can produce antibacterial active molecules or release free fatty acids to inhibit the growth of Streptococcus pneumoniae (20, 21).

### Inducing factors of microbial dysbiosis

Reduced respiratory microbial diversity and excessive proliferation of pathogenic bacteria are the common characteristics of microbial dysbiosis. The decreased diversity of the respiratory microbiota, the enrichment of pathogenic bacteria such as Pseudomonas aeruginosa and Staphylococcus aureus, and the reduced abundance of beneficial bacteria such as Prevotella, Lactobacillus and Corynebacterium are closely related to the occurrence and development of acute respiratory infections, asthma, bronchiectasis and other diseases (12, 22). Viral infection is a common inducement of respiratory microbial dysbiosis in children. Respiratory syncytial virus (RSV) and rhinovirus (RV) can increase the colonization rate of Streptococcus pneumoniae and Haemophilus influenzae in the upper respiratory tract by damaging the mucosal barrier and inhibiting the host immune response (23, 24). At the same time, the colonization of pathogenic bacteria also increases the host's susceptibility to viral infection. For example, the colonization of Streptococcus pneumoniae, Haemophilus influenzae and Moraxella catarrhalis in the nasopharynx of children is associated with an increased risk of RSV infection (23), nasopharyngeal colonized Haemophilus influenzae can aggravate the severe inflammatory response induced by RV infection by up-regulating the expression of Toll-like receptor 3 (TLR-3) and intercellular adhesion molecule (ICAM) on the surface of airway epithelial cells (24). Notably, the bacteria-virus interaction is not merely “synergistic pathogenicity”. For example, recent studies have confirmed that commensal bacteria such as Streptococcus pneumoniae can inhibit viral replication through “immune priming” or up-regulating the interferon signaling pathway (25). Zou et al. (26) reported that the enrichment of oral Streptococcus can inhibit influenza virus replication by enhancing the host innate immunity. In addition, factors such as altered immune function, nutritional status, age, gender, geographical region, diet, vaccination, antibiotic use, and environmental exposure (dust, fine particulate matter, allergens) can all induce changes or dysbiosis of the respiratory microbiome (5, 8, 12).

## Interaction between the respiratory Microbiota and host immunity

The respiratory microbiota can promote host immune maturation, immune tolerance and the formation of immune responses by regulating the balance of immune cells, enhancing mucosal barrier function, and producing metabolites (4, 19, 27–29). Pattaroni et al. (30) found that in the early life (especially the first 3 months), the airway bacterial community is highly synchronized with the development of the host local immunity, the flora composition changes significantly with age, and is associated with the expression of immune-related genes such as the interferon pathway and adaptive immune pathway. Asymptomatic pathogenic bacterial colonization in the neonatal URT has an immune stimulatory effect, and different pathogenic bacteria can induce specific inflammatory immune responses in the airway mucosa (31). Rosas-Salazar et al. (17) found that pathogens such as Klebsiella and Moraxella are positively correlated with the levels of type 2, type 3 and pro-inflammatory immune mediators. Pathogenic bacteria (such as Moraxella catarrhalis and Haemophilus influenzae) can induce a mixed Th1/Th2/Th17 inflammatory response, increase pro-inflammatory factors such as IL-1β and TNF-α, trigger chronic inflammation, and increase the risk of asthma in children in the future (17, 31). Nasopharyngeal microorganisms (such as Acinetobacter, Moraxella, Enterococcus, etc.) are associated with serum IgE levels, and may affect children's susceptibility to allergic respiratory diseases by regulating B cell production of IgE or T cell immune function (32). Beneficial nasopharyngeal bacteria (such as Corynebacterium and Dolosigranulum) can induce the production of cytokines such as IFN-*β* and IL-10 by activating the TLR3 signaling pathway, enhance immune defense, and reduce lung injury (17, 31). Jaeger et al. (33) reported that the mouse-adapted bacterium Bordetella colonized in the airway can up-regulate the expression of the antimicrobial peptide SLPI by inducing a Th17 immune response, thereby alleviating allergic airway inflammation.

In addition, short-chain fatty acids (SCFAs) such as acetate, propionate and butyrate among microbial metabolites can inhibit histone deacetylases, regulate gene expression and immune cell differentiation, and high levels of SCFAs can reduce the risk of asthma (34). SCFAs can also promote the generation of Tregs, act directly on T cells and dendritic cells, and improve microbial-induced pulmonary allergic inflammation (19). SCFAs can also activate β-interferon through the type Ⅰ interferon receptor signaling pathway to exert an anti-RSV infection effect (18).

## Interconnection between the airway and intestinal microbiota (gut-lung axis)

The intestinal microbiome can affect the host pulmonary diseases through immune regulation, metabolites and other pathways, forming a complex interaction network, and this regulatory mechanism is known as the “gut-lung axis”.

### Positive regulation of the gut-lung axis

Ngo et al. (35) reported that segmented filamentous bacteria (SFB) colonized in the intestine can protect mice from secondary infections such as Streptococcus pneumoniae, Haemophilus influenzae or Staphylococcus aureus after influenza A virus infection by epigenetically reprogramming alveolar macrophages (AMs). Metabolites and immune cells produced by the intestinal flora can reach the lungs through the blood circulation, regulate pulmonary immune function and affect the composition of the pulmonary flora (2), intestinal commensal microorganisms can activate the migration of type 2 innate lymphoid cells and type 3 innate lymphoid cells in the intestinal wall to the lungs, enhancing the host's resistance to respiratory infectious diseases (18). Ni et al. (36) confirmed that Lactobacillus fermentum exerts a lung protective effect by regulating sphingolipid metabolism through the gut-lung axis, thereby modulating the PI3K-AKT pathway. In addition, metabolites such as SCFAs (acetate, propionate, butyrate), bile acids and aromatic amino acid derivatives produced by the fermentation of intestinal anaerobes can reach the lungs through the blood circulation, regulate T cell differentiation, neutrophil migration and antimicrobial peptide expression by G protein-coupled receptors or inhibiting histone deacetylases, and exert anti-inflammatory and antiviral effects (37). The intestinal flora also plays a key role in abnormal immune responses such as asthma, and its metabolite SCFAs has a protective effect on neonatal asthma (1, 4), pregnant women's intake of a high-fiber diet can alter the composition of the intestinal flora, increase the production of SCFAs, and is expected to prevent the occurrence of asthma in offspring (19, 20).

### Reverse regulation of the gut-lung axis

Pathophysiological changes in the lungs (such as infection, inflammation, and floral disturbance) can reversely induce intestinal flora dysbiosis, intestinal barrier damage and intestinal immune disorder through the bidirectional interaction mechanism of the gut-lung axis. Components such as polysaccharides and lipopolysaccharides of the respiratory microbiome or viral infection can mediate the production of pulmonary interferons, thereby inducing intestinal inflammatory responses or intestinal microbial dysbiosis, and intestinal flora dysbiosis exacerbates pulmonary infection and inflammation through the gut-lung axis (2). For example, pulmonary respiratory syncytial virus infection can trigger pulmonary inflammation and destroy the development of Th17/Treg cells through a systemic inflammatory response, and at the same time induce the overexpression of RegⅢ*γ* through IL-22 to alter the composition of the intestinal flora, thereby leading to intestinal immune damage and floral dysbiosis (38). Zhang et al. (39) reported that influenza infection can disrupt the intestinal microbiota (increase in Enterobacteriaceae and decrease in Lactobacillaceae) and metabolites (abnormality of 2,3-dioxygenase 1), and supplementation with tryptophan and Lactobacillus can repair the gut-lung axis and alleviate influenza-induced lung injury. The core of the gut-lung axis regulation lies in the transorgan migration of immune cells and the systemic circulation of metabolites, which together form a microecology-immune regulatory network between the intestine and the lungs. At present, the specific regulatory mechanism of the gut-lung axis in childhood respiratory diseases remains to be further studied, which is expected to become a new target for the microecological treatment of childhood respiratory diseases.

## Association between the respiratory microbiota and childhood respiratory diseases

### Acute respiratory infection

Acute respiratory infections (ARI) such as bronchiolitis and pneumonia are major public health problems facing children worldwide, and the vast majority of pathogenic bacteria causing respiratory infections are common asymptomatic colonizers in the nasal cavity, nasopharynx and oropharynx (23). McCauley et al. (40) found that in children aged 4–7 years, the nasopharyngeal flora dominated by Moraxella is significantly associated with an increased risk of upper respiratory tract infection and sinusitis. The airway flora characteristics of acute respiratory infections in children are reduced diversity, with pathogenic bacteria such as Streptococcus pneumoniae, Haemophilus influenzae, Moraxella catarrhalis and Staphylococcus aureus as the dominant flora, while the number of beneficial bacteria such as Dolosigranulum and Corynebacterium is significantly reduced (18, 23, 40–42), as shown in Table 2. Reduced pulmonary microbial diversity is closely related to severe ARI and high mortality, children with the dominant flora of Enterococcus and Acinetobacter baumannii are more likely to be classified into the death and severe groups, while children with a higher abundance of Lactobacillaceae have a lower risk of developing severe ARI (43).

**Table 2:** Respiratory microbiome characteristics of common respiratory diseases in children.

Disease type	Core microbial characteristics and their relationship with diseases
Acute respiratory infection	Reduced diversity, enrichment of pathogenic bacteria such as Streptococcus pneumoniae, Haemophilus influenzae and Moraxella catarrhalis, and reduction of Dolosigranulum and Corynebacterium, the lungs with dominant ora such as Enterococcus and Acinetobacter baumannii indicate a more severe condition"change to `When the lung microbiota is dominated by genera such as Enterococcus and Acinetobacter baumannii, the clinical condition tends to be more severe
Bronchial asthma	Reduced diversity, enrichment of Moraxella and Haemophilus, and reduction of Corynebacterium and Lactobacillus, the enrichment of Haemophilus influenzae, Moraxella catarrhalis and other bacteria is associated with poor asthma control
Persistent bacterial bronchitis	Enrichment of pathogenic bacteria such as Streptococcus pneumoniae, Haemophilus influenzae, Moraxella catarrhalis, Staphylococcus aureus and Pseudomonas aeruginosa, the enrichment of Haemophilus influenzae is associated with disease progression
Bronchiectasis	Haemophilus influenzae is the most common, followed by Moraxella catarrhalis, Streptococcus pneumoniae, Pseudomonas aeruginosa and Staphylococcus aureus, the enrichment of Haemophilus influenzae and Pseudomonas aeruginosa is associated with recurrent infections and disease exacerbation
Cystic fibrosis	Diversity gradually decreases with age, and the abundance of pathogenic bacteria such as Pseudomonas aeruginosa and Staphylococcus aureus increases with age, patients with Pseudomonas aeruginosa, Staphylococcus aureus and other bacteria as the main flora are associated with significant decline in lung function, acute exacerbation and poor prognosis

### Bronchial asthma

Asthma is the most common chronic heterogeneous respiratory disease in children and adults, and upper respiratory flora dysbiosis is an important risk factor for it. Upper respiratory flora dysbiosis dominated by Haemophilus influenzae and Moraxella can increase the risk of wheezing in infants and young children by inducing a classic monocyte-related immune response (30), the dominance of flora such as Streptococcus pneumoniae, Haemophilus influenzae and Moraxella catarrhalis in the early infancy significantly increases the risk of pneumonia, asthma or wheezing in children, while Lactobacillus, Dolosigranulum and Corynebacterium are associated with a reduced risk of wheezing or asthma (22, 44, 45), as shown in Table 2.

The respiratory microbiota can affect the occurrence and development of asthma in children through multiple pathways such as microbial dysbiosis, metabolites, inflammatory responses and immune responses (46).The nasal flora diversity of asthmatic children is significantly reduced, dominated by Proteobacteria and Firmicutes, with a high abundance of Moraxella and Haemophilus, while the nasal flora of healthy children is also dominated by Proteobacteria and Firmicutes, it is richer in Staphylococcus and Corynebacterium (47). Floral diversity in early life can effectively reduce the risk of atopic diseases (including allergic diseases or asthma) in children or adults (22), especially the dominant flora dominated by Mycobacterium, which is associated with a reduced risk of asthma in the future (24). Proteobacteria can induce higher levels of pro-inflammatory cytokines such as IL-8 and TNF-α, significantly increase airway neutrophil infiltration, and simultaneously stimulate host dendritic cells to secrete IL-23 and IL-12, Bacteroidetes has an anti-inflammatory effect, which can inhibit the secretion of IL-12 induced by Haemophilus influenzae and alleviate the inflammatory response (48). The host's inflammatory response in turn promotes the growth of specific bacterial communities, further aggravating respiratory microbial dysbiosis (6, 44).

There are significant differences in the composition of the airway flora among asthmatic patients with different severity and types (22).The enrichment of Sphingomonas in the airway flora is associated with airway eosinophilia and obvious airway hyperresponsiveness (49).Haemophilus influenzae can activate macrophages and induce glucocorticoid resistance in asthmatic patients (6), the enrichment of Haemophilus influenzae and Moraxella catarrhalis is also closely related to severe airway obstruction, high neutrophilic airway inflammation and poor asthma control (6, 44).

### Persistent bacterial bronchitis

Persistent bacterial bronchitis (PBB) in children is one of the common causes of chronic wet cough in children under 5 years old (50), and severe acute lower respiratory tract infection in early childhood is associated with the occurrence of PBB (51). PBB and bronchiectasis share common underlying pathophysiological mechanisms, including chronic wet cough, mucociliary clearance dysfunction, endobronchial bacterial infection and neutrophilic airway inflammation (50, 51). In the bronchoalveolar lavage fluid (BALF) of children with PBB, pathogenic bacteria such as Streptococcus pneumoniae, Haemophilus influenzae, Moraxella catarrhalis, Staphylococcus aureus and Pseudomonas aeruginosa are enriched (52, 53), as shown in Table 2. The diversity and dominant flora of the airway microbiome are closely related to the prognosis of children with PBB. When children have recurrent Haemophilus influenzae infection, vigilance should be raised against the possibility of progression to bronchiectasis (54), Ruffles et al. (55) reported that about 9.6% of PBB patients are complicated with bronchiectasis, and recurrent persistent bacterial bronchitis in the first year of follow-up and the enrichment of Haemophilus influenzae in bronchoalveolar lavage fluid are important predictive factors for bronchiectasis.

### Bronchiectasis

Bronchiectasis in children is a heterogeneous respiratory disease characterized by permanent airway dilation (53). Recurrent respiratory tract infections in childhood that damage the airway are the main inducement (56), and the pathogenesis mainly includes host factors (genetic factors, primary immunodeficiency, primary ciliary dyskinesia, etc.) and environmental factors (pathogenic bacterial infection, environmental pollutants) (56). The impaired mucociliary clearance ability of the airway mucosa in patients with bronchiectasis is conducive to the colonization of pathogenic bacteria, thereby triggering the non-specific immune response of the host (2). In recent years, studies have shown that the decreased flora diversity and enrichment of pathogenic bacteria in the BALF of children with bronchiectasis are associated with recurrent infections and disease exacerbation (2, 56). There are differences in the respiratory microbiome of bronchiectasis between adults and children. Rogers et al. (57) reported that the detection rate of Pseudomonas aeruginosa in adult patients with bronchiectasis is as high as 24%, but it is relatively low in pediatric patients. Moriki et al. (58) conducted a retrospective analysis of 50 children with bronchiectasis and found that Haemophilus influenzae is the most common in bronchial BALF (28.0%), followed by Moraxella catarrhalis, Streptococcus pneumoniae, Pseudomonas aeruginosa, Staphylococcus aureus and so on. Airway-colonized pathogenic bacteria such as Pseudomonas aeruginosa, Haemophilus influenzae and Streptococcus pneumoniae are associated with neutrophilic inflammatory response, acute exacerbation, decreased lung function and poor prognosis in children (2, 57, 58), as shown in Table 2. The synergistic pathogenic effect of fungi and bacteria is an important mechanism for the pathogenesis of bronchiectasis. A study on adults reported fungal infections in the airways of patients with non-cystic fibrosis bronchiectasis, with Candida albicans (21.8%) being the most common (59). There are few research reports on the pathogenesis of bronchiectasis associated with fungi in children. Fungal infections in children are mainly caused by Aspergillus and Candida, which are often closely related to airway structural damage, immune disorder, long-term use of antibiotics or hormones (60). In addition, the virus detection rate at the initial onset of bronchiectasis in children is 48%–53%, and common viruses include human rhinovirus, human coronavirus, influenza virus, parainfluenza virus, adenovirus and respiratory syncytial virus (61). At present, the interaction between viruses and fungi in the pathogenesis of bronchiectasis in children remains unclear and needs further study.

### Cystic fibrosis

Cystic fibrosis (CF) is an autosomal recessive monogenic disease caused by mutations in the cystic fibrosis transmembrane conductance regulator (CFTR) gene (2). There are significant differences in the composition of the pulmonary flora between CF patients and healthy people, characterized by relatively low microbial diversity and dominated by Staphylococcus and Pseudomonas (62, 63). Pseudomonas aeruginosa colonization is closely related to the severity of pulmonary involvement, deterioration of lung function, aggravation of airway inflammation and increased activity of neutrophil elastase (64, 65).

The pulmonary microbial diversity of children with CF gradually decreases with age, and the abundance of pathogenic bacteria such as Pseudomonas aeruginosa and Staphylococcus aureus increases with age (63): children under 2 years old are dominated by Haemophilus, children aged 2–10 years have Staphylococcus aureus and Stenotrophomonas maltophilia as the dominant bacteria, and older patients have a higher proportion of Pseudomonas aeruginosa (66), as shown in Table 2. CF patients with pathogenic bacteria such as Pseudomonas aeruginosa and Staphylococcus aureus as the main flora have a significant decline in lung function, while patients with better lung function have a higher proportion of obligate anaerobes such as Prevotella and Porphyromonas (67). Pseudomonas aeruginosa can also cause chronic infections, leading to the colonization of various pathogenic bacteria, acute exacerbation of lung injury, and ultimately premature death of patients (2, 8).

CFTR modulator therapy is a cutting-edge direction in CF treatment. This therapy can regulate the pulmonary microecology by repairing the function of the CFTR protein and improving the airway mucociliary clearance ability (67). CFTR modulators can significantly reduce the colonization rate of pathogenic bacteria such as Pseudomonas aeruginosa and Staphylococcus aureus in CF patients, increase pulmonary microbial diversity, and restore the abundance of beneficial bacteria. Its regulatory effect on the respiratory microbiome has become an important research direction for the microecological treatment of CF (68). In addition, the mycobiome and virome also play a key role in the pathogenesis of CF: rhinovirus infection can promote the colonization and reproduction of pathogenic bacteria in CF patients (2). The number of Candida albicans in the lungs of CF patients increases, and it often grows symbiotically with Pseudomonas aeruginosa (2), and the two synergistically aggravate airway inflammation and lung function damage.

## Therapeutic strategies related to the respiratory microbiota

In recent years, in-depth research on the respiratory microbiome has prompted a growing interest in airway microbiome engineering—, i.e., the intentional manipulation of these microorganisms to restore floral balance, enhance respiratory mucosal defense function, and treat respiratory diseases (69). The respiratory microbiota is an important target for the treatment of childhood respiratory diseases. The traditional therapeutic strategy is mainly based on antibiotics. Combined with the cutting-edge research in recent years, novel therapeutic strategies such as probiotic therapy, phage therapy, and antimicrobial peptide (AMP) therapy have gradually become research hotspots (as shown in Table 3), providing new therapeutic directions for respiratory diseases related to multidrug-resistant bacterial infections and microecological dysbiosis.

**Table 3:** Research on therapeutic strategies related to the respiratory microbiota.

Therapeutic method	Latest relevant research
Probiotics via throat spray, nasal spray or oral administration	Zhang et al. (70) reported that inhalation of L. plantarum and L. murinus can activate V*γ*4 γ*δ* T cells to secrete IL-17A, which is conducive to reducing the host's susceptibility to infection with Staphylococcus aureus and Pseudomonas aeruginosa.
	He et al. (71) reported that oral administration of Lactobacillus curvatus IM01 can target and reshape the intestinal flora structure, which is conducive to the enrichment of beneficial bacteria such as Lactobacillus and Olfacterium, restore the levels of key immune-regulating metabolites such as indole lactic acid and choline, and alleviate airway inflammation and allergic symptoms.
Rational application of antibiotics	Broderick et al. (54) found that the diversity of the airway microbiota did not decrease but slightly increased after antibiotic treatment in children with bronchiectasis during acute exacerbation.
	Raita et al. (76) found that systemic antibiotic exposure in the early infancy (0–2 months) can alter the age-related evolutionary trajectory of the nasal microbiota, replacing the dominance of beneficial commensal bacteria (such as Bacteroides thetaiotaomicron) with potential pathogenic bacteria (such as Haemophilus).
Phage therapy	Chen et al. (81) reported a novel bacteriophage phiTH1 and its encoded depolymerase Dop5, which can enhance the host immune clearance ability and alleviate the inflammatory response by specifically degrading the capsular polysaccharide of Klebsiella pneumoniae type K30.
	Weiner et al. (82) conducted a study on nebulized phage cocktail therapy BX004-A for chronic Pseudomonas aeruginosa infection in CF patients, and found that BX004-A can significantly reduce the number of Pseudomonas aeruginosa colonies in the sputum of patients and increase the flora diversity.
Antimicrobial peptide (AMP) therapy	Ben et al. (85) found that a D,L-K6L9p peptide exerts potent antibacterial and anti-biofilm activity against Pseudomonas aeruginosa infection in CF patients.

### Probiotics

In recent years, a number of studies have reported that respiratory and intestinal commensal bacteria and their metabolites play an important role in host anti-infection, immune regulation and inflammatory response regulation. At present, there are many reports on the curative effect of Lactobacillus, but Lactobacillus has significant strain specificity. For example, Zhang et al. (70) reported that inhalation of pulmonary commensal Lactobacillus, especially L. plantarum and L. murinus, can activate Vγ4 γδ T cells to secrete IL-17A, thereby reducing the host's susceptibility to infection with Staphylococcus aureus and Pseudomonas aeruginosa. He et al. (71) reported that oral administration of Lactobacillus curvatus IM01 can target and reshape the intestinal flora structure, which is conducive to the enrichment of beneficial bacteria such as Lactobacillus and Olfacterium, restore the levels of key immune-regulating metabolites such as indole lactic acid and choline, promote the differentiation of CD4⁺ T cells into Foxp3⁺ regulatory T cells (Tregs), inhibit Th2-type immune response and inflammatory cell infiltration, and ultimately alleviate airway inflammation and allergic symptoms. A clinical trial of a live Lactobacillus throat spray in healthy adults showed that specific Lactobacillus strains have immune-stimulating and anti-respiratory viral infection effects (72). Koidl et al. (73) reported that Staphylococcus epidermidis can effectively alleviate allergic symptoms in mice and reduce eosinophil infiltration in the nasal mucosa by regulating the local nasal microecology and immune balance. Some commensal bacteria also have the ability to destroy biofilm matrices. For example, Staphylococcus epidermidis can not only destroy the biofilm of Staphylococcus aureus but also increase the sensitivity of Staphylococcus aureus to host antimicrobial peptides (24). At the same time, Mazzolini et al. (74) found that a live genetically engineered strain of Mycoplasma pneumoniae has a significant therapeutic effect on ventilator-associated pneumonia and tracheal intubation biofilm infection caused by clinical multidrug-resistant Pseudomonas aeruginosa. In addition, SCFAs produced by commensal bacteria can effectively alleviate allergic airway inflammation (24). As metabolites of probiotics, SCFAs can exert immunoregulatory and anti-inflammatory effects through oral or airway administration, and become potential therapeutic drugs for childhood respiratory diseases.

### Rational application of antibiotics

Antibiotics have a dual role in the treatment of respiratory diseases. Rational use can reconstruct the normal microbial flora and significantly reduce the colonization and infection of pathogenic bacteria, especially infections caused by multidrug-resistant bacteria (2). Broderick et al. (54) found that the diversity of the airway microbiota did not decrease but slightly increased after antibiotic treatment in children with bronchiectasis during acute exacerbation, which may be related to the recovery of low-abundance microorganisms after antibiotic treatment inhibits the dominant pathogenic bacteria. Henares et al. (75) also found that oropharyngeal Streptococcus pneumoniae decreased in children with invasive pneumococcal disease after antibiotic treatment, and oral bacteria and hospital-acquired bacteria may achieve short-term nasopharyngeal floral reconstruction through oral flora migration and the expansion of low-abundance flora. Abusive use of antibiotics will reduce the diversity of the respiratory microbiota and destroy the composition of the airway flora. Raita et al. (76) found that systemic antibiotic exposure in the early infancy (0–2 months) can alter the age-related evolutionary trajectory of the nasal microbiota, replacing the dominance of beneficial commensal bacteria (such as Bacteroides thetaiotaomicron) with potential pathogenic bacteria (such as Haemophilus), which is associated with an increased risk of respiratory tract infections, recurrent wheezing and asthma in childhood. A study on the effect of anaerobic antibacterial spectrum on the airway microbiome diversity and lung function of children with cystic fibrosis found that broad-spectrum anti-anaerobic antibiotics cause more significant and longer-lasting damage to the airway microbiome of CF children than narrow-spectrum antibiotics (77). Pettigrew et al. (78) also found that for children with community-acquired pneumonia, compared with 5-day β-lactam antibiotic treatment and 10-day standard treatment, the abundance and duration of resistance genes in children receiving 10-day standard treatment were significantly higher. Therefore, clinical practice should be alert to the overuse of antibiotics, strictly control the duration of medication, and reduce the damage to airway commensal bacteria and the emergence of antibiotic-resistant bacteria.

### Phage therapy

Bacteriophages are a class of viruses that specifically infect bacteria, which can accurately target and lyse pathogenic bacteria without easily destroying the balance of the normal flora, providing a novel therapeutic strategy for respiratory diseases related to multidrug-resistant bacterial infections and floral dysbiosis (79, 80). In diseases such as bronchiectasis and CF, the treatment of infections caused by multidrug-resistant bacteria such as Pseudomonas aeruginosa and Staphylococcus aureus is a worldwide problem. Phage therapy can specifically eliminate such pathogenic bacteria and reduce infection recurrence and acute exacerbation (81). For example, Chen et al. (81) reported a novel bacteriophage phiTH1 and its encoded depolymerase Dop5. Dop5 can enhance the host immune clearance ability and alleviate the inflammatory response by specifically degrading the capsular polysaccharide of Klebsiella pneumoniae type K30, showing significant therapeutic potential in a mouse model of inhalational pneumonia. A nebulized phage cocktail therapy BX004-A for chronic Pseudomonas aeruginosa infection in CF patients found that the number of Pseudomonas aeruginosa colonies in the sputum of patients decreased significantly and the flora diversity increased during BX004-A treatment (82). Lebeaux et al. (83) reported the first successful case of phage therapy for pan-drug-resistant Achromobacter xylosoxidans infection in a lung transplant patient, and confirmed that phage therapy is effective in the treatment of multidrug-resistant bacterial infections with good tolerance. At present, there are few studies on phage therapy in the field of childhood respiratory diseases. In the future, it is necessary to focus on exploring the targeting, safety and combination therapy regimens of bacteriophages (such as combined use with antibiotics and probiotics) to provide more effective treatment options for pediatric patients.

### Antimicrobial peptide (AMP) therapy

Antimicrobial peptides (AMPs) are key components of the innate immune system of various organisms including humans. AMPs also play an important role in a variety of physiological processes, including inducing angiogenesis, promoting wound healing, and regulating immune responses (84). AMPs can regulate the microbiome and resist microbial infections of the skin, lungs and gastrointestinal tract (84). They show great potential in the treatment of the respiratory microbiome in children, and are especially suitable for diseases related to drug-resistant bacterial infections and floral dysbiosis. For example, CFTR gene mutation leads to viscous airway mucus, which is prone to induce the colonization of bacteria such as Pseudomonas aeruginosa to form biofilms, thereby causing chronic pulmonary infection. Ben et al. (85) found that a D,L-K6L9p peptide exerts potent antibacterial and anti-biofilm activity against Pseudomonas aeruginosa infection in CF patients, and it can also resist degradation by CF sputum proteases and does not induce bacterial resistance. Another study reported that a humanized DNABII-targeted monoclonal antibody can convert drug-resistant bacteria (nontypeable Haemophilus influenzae, methicillin-resistant Staphylococcus aureus) in biofilms into the NRel phenotype, which is significantly sensitive to common respiratory antimicrobial peptides or those produced by neutrophils (PMNs) and can be more efficiently killed (86). At present, AMPs have problems such as short half-life, partial toxic and side effects, and incompletely clear mechanism of action, which need further in-depth study to lay a foundation for their clinical application.

## Conclusion

A healthy respiratory microbiota plays an important role in lung development and immune maturation in children. The imbalance among the host, environment and microbiome is an important factor leading to acute and chronic respiratory inflammation, mucosal dysfunction and related diseases in children. In recent years, relevant research on the respiratory microbiome has deepened people's understanding of commensal flora as a target for targeted therapy of respiratory diseases, but the research on the respiratory microbiome in children is still in its infancy, and many problems remain to be solved: first, the lack of baseline data on the respiratory microbiome (especially the lower respiratory tract) of healthy children, and the normal reference range of the respiratory microbiome of children of different ages and regions has not been established due to the difficulty in sample collection, second, the causal relationship between the microbiome and respiratory diseases has not been fully clarified, third, there are few pediatric clinical studies on novel microecological therapeutic strategies, and their targeting, safety and effectiveness need further exploration. As a novel therapeutic strategy, microecological therapy has the advantages of strong targeting, few side effects and low tendency to produce drug resistance, and is expected to become a new direction for the treatment of childhood respiratory diseases.
